# Measuring in-hospital quality multidimensionally by integrating patients’, kin’s and healthcare professionals’ perspectives: development and validation of the FlaQuM-Quickscan

**DOI:** 10.1186/s12913-023-10349-2

**Published:** 2023-12-16

**Authors:** Fien Claessens, Deborah Seys, Charlotte Van der Auwera, Anneke Jans, Eva Marie Castro, Laura Jacobs, Dirk De Ridder, Luk Bruyneel, Zita Leenaerts, Astrid Van Wilder, Jonas Brouwers, Peter Lachman, Kris Vanhaecht, The FlaQuM Research Group, Ann Baeyens, Filip Bouckaert, Isabel De Brauwer, Mieke De Medts, Kathleen De Sutter, Elke De Troy, Eddy Delporte, Nina Donvil, Guy Hans, Lieven Hoebrekx, Sarah Loubele, Frank Martens, Tinneke Mues, Kristin Muller, Bart Pardon, Karolien Pennewaert, Ingrid Roosen, Kristin Muller, Inge Sedeijn, Frank Staelens, Sandra Stevens, Goedele Tavernier, Birte Theunissen, Ines Van Giel, Els Van Zele, Koen Vanachter, Jef Vanderoost, Dirk Vanrenterghem, Nele Vanstraelen, Gerda Verheyden, Joan Vlayen, Annick Wauters, Sofie Wijnen

**Affiliations:** 1 Leuven Institute for Healthcare Policy, Department of Public Health and Primary Care, KU Leuven – University of Leuven, Leuven, Belgium; 2 Department of Quality Management, Sint-Trudo Ziekenhuis, Sint-Truiden, Belgium; 3 Department of Quality Management, Regionaal Ziekenhuis Heilig Hart Tienen, Tienen, Belgium; 4 Department of Quality Management, University Hospitals Leuven, Leuven, Belgium; 5 Department of Orthopaedics, University Hospitals Leuven, Leuven, Belgium; 6 Lead Faculty Quality Improvement Programme- Royal College of Physicians of Ireland, Dublin, Ireland

**Keywords:** Quality assurance, Health care, Psychometrics, Health care survey, Patient-centered care, Family, Health personnel, Caregivers

## Abstract

**Background:**

Measuring quality is essential to drive improvement initiatives in hospitals. An instrument that measures healthcare quality multidimensionally and integrates patients’, kin’s and professionals’ perspectives is lacking. We aimed to develop and validate an instrument to measure healthcare quality multidimensionally from a multistakeholder perspective.

**Methods:**

A multi-method approach started by establishing content and face validity, followed by a multi-centre study in 17 Flemish (Belgian) hospitals to assess construct validity through confirmatory factor analysis, criterion validity through determining Pearson’s correlations and reliability through Cronbach’s alpha measurement. The instrument FlaQuM-Quickscan measures ‘Healthcare quality for patients and kin’ (part 1) and ‘Healthcare quality for professionals’ (part 2). This bipartite instrument mirrors 15 quality items and 3 general items (the overall quality score, recommendation score and intention-to-stay score). A process evaluation was organised to identify effective strategies in instrument distribution by conducting semi-structured interviews with quality managers.

**Results:**

By involving experts in the development of quality items and through pilot testing by a multi-stakeholder group, the content and face validity of instrument items was ensured. In total, 13,615 respondents (5,891 Patients/kin and 7,724 Professionals) completed the FlaQuM-Quickscan. Confirmatory factor analyses showed good to very good fit and correlations supported the associations between the quality items and general items for both instrument parts. Cronbach’s alphas supported the internal consistency. The process evaluation revealed that supportive technical structures and approaching respondents individually were effective strategies to distribute the instrument.

**Conclusions:**

The FlaQuM-Quickscan is a valid instrument to measure healthcare quality experiences multidimensionally from an integrated multistakeholder perspective. This new instrument offers unique and detailed data to design sustainable quality management systems in hospitals. Based on these data, hospital management and policymakers can set quality priorities for patients’, kin’s and professionals’ care. Future research should investigate the transferability to other healthcare systems and examine between-stakeholders and between-hospitals variation.

**Supplementary Information:**

The online version contains supplementary material available at 10.1186/s12913-023-10349-2.

## Background

In the past 20 years, healthcare quality initiatives were mainly related to six quality domains as defined by the Institute of Medicine (IOM): patient-centredness, timeliness, efficiency, effectiveness, safety and equity [1]. Recently, Lachman and colleagues reflected on the relevance of IOM’s quality domains and suggested a multidimensional quality model that includes new domains. The revised domains reflect the changing worldview of quality management [2, 3], such as ecology [4] and transparency [5]. Lachman's new quality model extends the domain of person-centredness by recognising the patient’s kin and healthcare professionals as persons with fundamental needs embodied in every other quality domain. Kin involvement is increasingly being seen as an individual component of quality initiatives that can lead to improved patient outcomes [6–8]. Emphasis is placed on including their experiences as an important knowledge source for quality purposes [9]. Moreover, research has shown that quality of care (QoC) and patient safety are related to professionals’ characteristics, such as a negative association with burnout [10, 11], and that their working environment should be monitored [12]. The incorporation of care for professionals has been reinforced by the transition from the Triple Aim to the Quintuple Aim for improving healthcare, with an emphasis on healthcare equity [13]. To conclude, integrating experiential knowledge of patients, kin and professionals about QoC for patients and kin as well as for professionals is recognised as important considering the trend towards value-based, co-produced quality management systems.

In order to effectively co-produce an organisation-wide quality management system, it is essential to approach QoC multidimensionally and integrate it from a multistakeholder perspective [14–16]. Many instruments have been developed to measure experiences of QoC [12, 17–26] and quantifying them has become widespread [27]. Nevertheless, existing instruments have focused on a particular stage of a patients’ hospital journey from admission [17] to hospital discharge [18], on a specific disease, e.g. in cardiology care [19], on certain quality domains, e.g. such as communication and coordination of care [20] or on including only patients [21, 22], kin [23] or professionals [12, 24–26]. An instrument that captures organisation-wide experiences would provide a comprehensive healthcare quality assessment whose results can catalyse meso- and macro-level quality management, such as prioritising quality improvement efforts based on multistakeholder experiences. Such an instrument, that encompasses all quality domains [2] and integrates patients’, kin’s and professionals’ perspectives on these domains, is currently lacking. The absence of experiences from other quality domains, such as Lachman’s core values and catalysts, which has been highlighted as desired quality outcomes in previous research [28–32], leads currently to a non-comprehensive view on QoC in hospitals. In addition, blind spots from other stakeholders’ experiences prevent hospitals from creating a quality management system that creates value for all. Measuring QoC multidimensionally from a multistakeholder perspective is fundamental for hospitals to gain a deeper understanding of experiences. Though, no studies have so far constructed a bipartite, organisation-wide instrument measuring both healthcare quality for ‘patients and kin’ and how the organisation cares for its ‘professionals’ in a methodologically sound way that involved patients, kin and professionals. Results of such a validated instrument will facilitate co-production of a sustainable, organisation-wide quality management system in which all stakeholders’ values are central. In conclusion, we need a valid instrument encompassing quality multidimensionally in terms of care for patients, kin and for professionals and integrating multistakeholder perspectives, i.e. with patients, kin and professionals as key stakeholders in quality management. To address this research gap, we aimed to develop and validate an instrument to measure experiences of healthcare quality multidimensionally from a multistakeholder perspective.

## Methods

### Design

**Fig. 1:**
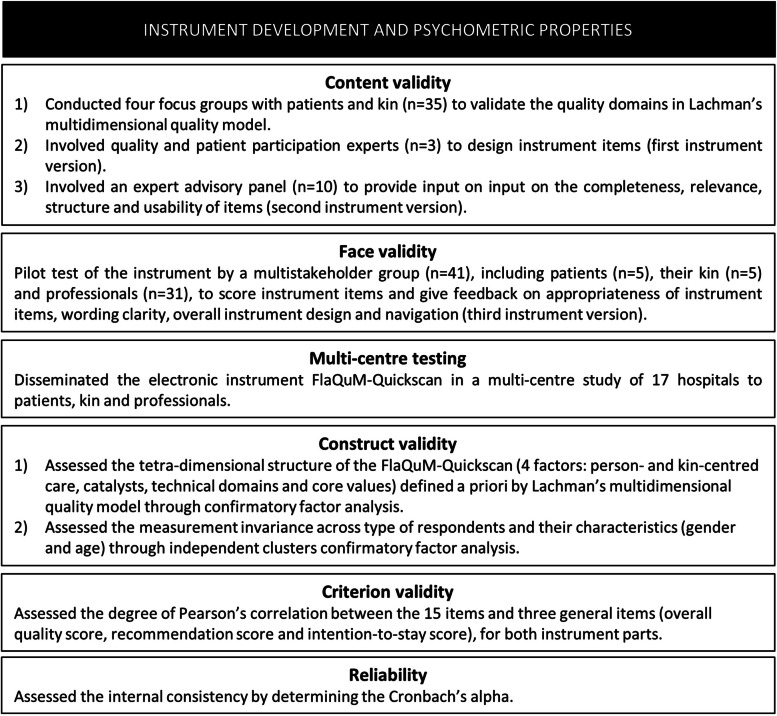
Instrument development and assessment of psychometric properties

A multi-method approach was used to develop and validate a rigorous instrument [33]. Development started by establishing the content and face validity, followed by testing the construct and criterion validity as well as the reliability using a cross-sectional survey design in 17 Flemish (Belgian) hospitals (Fig. 1). Data were collected between May 2021 and June 2022 via an online survey, the Flanders Quality Model (FlaQuM)-Quickscan. A parallel process evaluation was organised to identify effective strategies in instrument distribution by conducting semi-structured interviews with healthcare quality managers [34].

## Instrument development and psychometric properties

### Content validity

Content validity, also known as theoretical analysis, referred to the adequacy with which a measure assesses the domain of interest, i.e. that the items capture the relevant experience of the target population being examined [33]. First, our research group conducted four focus groups with patients and kin (n = 35 in total, n_patients_ = 23 and n_kin_ = 12) to gain a deeper understanding of key attributes of QoC relevant to them [35]. ‘Kin’ refers to the wider social construct around the people involved in receiving and providing care [2]. Kin is also known as caregivers, as used in other international publications [36, 37]. Caregiver refers to someone who takes care of a person who is young, old, ill, or disabled, i.e. having an illness, injury, or condition that makes it difficult for them to do some things that other people do, either as a family member or friend, or as a job [38]. As the word ‘kin’ is used in Lachman’s original multidimensional quality model [2], this term is also used in this manuscript to decrease the risk of confusion between the instrument and Lachman’s model [2]. Focus group results were mainly related to the quality domains ‘Partnership and co-production’, ‘Dignity and respect’ and ‘Effectiveness’. Technical quality domains were linked to organisational aspects of care in terms of staffing levels and time. A theory-based, inductive interpretation of patients’ and kin’s experiential knowledge during these focus groups resulted in the validation of Lachman’s multidimensional quality model. This model, that was developed by QoC experts using deductive reasoning based on expertise of healthcare researchers and professionals’ knowledge over the past 20 years, served as a conceptual framework for the development of the instrument [2]. Second, to design instrument items, three quality and patient participation experts (two are postdoctoral fellows, one with specific expertise in patient participation and empowerment and one with additional experience as member of a patient association in a Flemish hospital, and one is staff member specialised in patient participation in a Flemish hospital), were involved to consider content relevance of instrument items and to ensure operational ‘fit’ with the theoretical meaning of quality domains. An expert advisory panel (*n* = 10), consisting of the instrument’s target population (patients, kin and different types of professionals) provided input on the completeness, relevance, structure and usability of items. Based on their feedback, instrument items were revised.

### Face validity

Face validity, which is defined as the appropriateness of instrument items to the intended construct [33], was obtained through a pilot test by a multistakeholder group (*n* = 41), including patients (*n* = 5), their kin (*n* = 5) and professionals (*n* = 31). The latter were hospital board members (*n* = 4), executives (*n* = 11), healthcare quality managers (*n* = 4), physicians (*n* = 6), nurses (*n* = 5) and medical secretary (*n* = 1). In addition to scoring instrument items, they were asked to give feedback about appropriateness of instrument items, wording clarity, overall instrument design and navigation. The pilot test results were used to develop an updated version of the instrument.

### Description and scoring of the instrument

The instrument, hereinafter referred to as FlaQuM-Quickscan, is designed to mirror patients’, kin’s and professionals’ experiences of QoC through two parts that measure identical quality domains from different care perspectives [See Additional file 1]. The first part aims to explore perspectives on ‘Healthcare quality for patients and kin’, the second part on ‘Healthcare quality for professionals’, i.e. how the hospital cares for their professionals. Patients, kin and professionals were asked to complete both instrument parts. Each part includes 15 items, measuring exactly the same domains, i.e. those of the multidimensional quality model [2], three general items, two of which (the overall quality score and recommendation score) are based on international [39] and Belgian questionnaires [40] and one (the intention-to-stay score) was included because of the importance of this topic in the healthcare landscape and the current shortage of professionals, and sociodemographic questions. The 15 items reflecting quality domains were divided into four subscales: person- and kin-centred care (2 items), catalysts (3 items), technical domains (6 items) and core values (4 items). Each item was rated on a 11-point Likert-type scale reflecting the respondent’s level of disagreement or agreement with the item statement [score from “0” (strongly disagree) to “10” (strongly agree)]. The three general items started with the overall quality assessment of received care (in part 1) and the overall quality assessment of the hospital as employer (in part 2) [score from “0” (worst possible quality) to “10” (best possible quality)]. The second general item concerned the willingness to recommend the hospital to family and friends for receiving care (in part 1) or to work as an employee (in part 2) [score from “0” (definitely no) to “10” (definitely yes)]. The last general item reflected on respondents’ intention-to-stay in the next year to receive care (in part 1) or to work as employee (in part 2) [score from “0” (definitely no) to “10” (definitely yes)]. Demographic items included respondent groups (patients, kin or different professional groups), gender and age. The instrument language was Dutch.

### Multi-centre testing: setting and participants

This study took place in a convenience sample of 17 hospitals in Flanders (Belgium), which are implementing a new Flanders Quality Model (FlaQuM). FlaQuM focuses on developing a sustainable quality management system and encompasses 3 pillars: 1) “thinking” based on a quality vision model [2]; 2) “doing” by focusing on the implementation of a co-creation roadmap [14] and 3) “learning and innovating” from social capital in inter-hospital collaboratives [41]. The FlaQuM-Quickscan is part of pillar 1. Patients and their kin who had a consultation, treatment or admission in one of the included hospitals were invited to complete the FlaQuM-Quickscan. Dutch-speaking participants (patients, kin and professionals) of at least 18 years old were invited to complete the FlaQuM-Quickscan online. A FlaQuM Coordinator, i.e. the local healthcare quality manager, for each hospital was responsible for distributing the survey link for their hospital. The link to the electronic survey was provided by the University of Leuven and all the response data flowed to the university database. Each hospital invited patients, kin, or its professional staff to complete the survey, whether by way of e-mail, website, or a limited, local hospital portal. Only fully completed instruments (part 1 and part 2) were included in this study. In line with recommendations, a minimum sample size of 300 patients and kin and 300 professionals was considered acceptable for testing the FlaQuM-Quickscan validation [42].

### Descriptive statistics

Descriptive analyses of sociodemographic data delineated frequencies across type of respondents and their characteristics (gender and age). Descriptive analyses for each of the 15 items reflecting quality domains and for the three general items included average, percentage distribution of scores on the 11-point Likert scale and percentage of scores between 0–5, between 6–7 and between 8–10. The Kolmogorov–Smirnov test, a test to assess whether two samples have the same statistical distribution, was used to compare percentage distributions of scores on the 11-point Likert scale between patients/kin and professionals. The t-test, a test to assess differences between two independent groups, was used to compare averages of the 15 items and three general items scored by patients/kin and professionals. The level of significance was set to *p* < 0.05. The descriptive analyses were generated using the SAS software, Version 9.4 of the SAS System for Windows.

### Construct validity

First, confirmatory factor analysis (CFA) was performed to evaluate the tetra-dimensional structure of the FlaQuM-Quickscan (person- and kin-centred care, catalysts, technical domains and core values) defined a priori by the multidimensional quality model [2]. We assessed whether the hypothesised subscales of part 1 and 2 are conceptualized as such by patients, kin and professionals. Second, independent clusters (ICM)-CFA was used to assess measurement invariance across type of respondents and their characteristics. By doing so, the model fit across groups of respondents could be evaluated. To start, model fit was assessed in each group by conducting single-group CFA to investigate whether the established dimensionality of the instrument fit the two stakeholder groups separately: patients/kin and professionals [43]. Next, multiple group ICM-CFA was conducted to assess various types of invariance [44]. Configural invariance relates to showing the same pattern of associations between items and factors and the same number of factors. Factor loadings and thresholds are free across groups. Evidence of scalar invariance is a requirement for drawing meaningful comparisons across groups [44]. All items were continuous for all described steps. For multiple‐group ICM‐CFA, weighted least squares estimation with delta parameterization was used. In multiple‐group analyses, factor variances and latent means were fixed to be 1 and 0, respectively, for identification purposes [45]. Model fit evaluation was based on internationally recognised cut-off criteria [46] and Chen’s [47] allowed changes in fit indices when studying invariance for the Comparative Fit Index (CFI) (ranges between 0 and 1; reasonable if > 0.90 and very good if > 0.95), the Tucker–Lewis index (TLI) [48] (ranges between 0 and 1; reasonable if > 0.90 and very good if > 0.95), and the Root Mean Square Error of Approximation (RMSEA) [49] (ranges between 0 and 1; good fit if < 0.1). Mplus version 7.1 was used to estimate factor analytic models [45].

### Criterion validity

Criterion validity, defined as the degree of a relationship between a given test score and performance on another measure [33], was assessed by determining the degree of Pearson’s correlation between the 15-item instrument and the three general items (overall quality score, recommendation score and intention-to-stay score) for each instrument part. Coefficients exceeding *r* = *0.3* were considered as meaningful [50]. As no other instrument was available to measure patients’, kin’s and professionals’ experiences of QoC as defined by Lachman’s multidimensional model, scores on general items were treated as a substitute for a gold standard with which the instrument items were correlated*.* The general items have been found to relate well to quality domains [51, 52]. The overall quality score and recommendation score are also used for public reporting of patient experiences via the Hospital Consumer Assessment of Healthcare Providers and Systems (HCAHPS) in the United States [39] and in Belgium [40]. The intention-to-stay score received international attention because of the increasing shortage of healthcare professionals [53] and is used in Belgium as a smoke signal for policymakers and managers [54].

### Reliability

To obtain reliability for the FlaQuM-Quickscan, the internal consistency was measured using the Cronbach’s alpha for both instrument parts, the subscales and for both stakeholder groups (patients/kin and professionals), with a coefficient ≥ 0.7 considered to be good [33].

## Process evaluation

The process evaluation aimed to identify effective strategies to communicate the FlaQuM-Quickscan, to distribute it hospital-wide and to motivate patients, kin and professionals to complete. This evaluation started in three pilot hospitals by taking observation notes from all activities related to its aim. Qualitative, thematic analysis of notes led to lessons learned for other hospitals. Based on these lessons, a topic list and interview guide were developed to conduct semi-structured interviews with healthcare quality managers of the 17 included hospitals. This manager leads the overall coordination of instrument distribution in their hospital. All interviews were audio recorded. The rapid identification of themes from audio recordings (RITA) was used as a first data analysis step [55]. RITA allows for expeditious identification of themes in qualitative data while minimizing the loss of information. Next, thematic analysis was used to inductively analyse the data and to gradually develop and refine insights into effective strategies [56]. Research team (all authors) discussions enabled interpretation of the data and identification of strategies. The data analysis was performed in MS Excel.

## Ethical considerations

Ethical approval was obtained from all local ethics committees of participating hospitals. All respondents (focus groups, FlaQuM-Quickscan and process evaluation) provided informed consent. All methods were carried out in accordance with the Declaration of Helsinki guidelines and regulations.

## Results

### Developed instrument ‘FlaQuM-Quickscan’

By involving experts in the development of quality items and through pilot testing by a multi-stakeholder group, the content and face validity of the instrument and instrument items was ensured. During the development steps, the number of items remained the same, but the wording in item statements was adjusted based on feedback. The FlaQuM-Quickscan contains two parts (part 1 ‘Healthcare quality for patients and kin’ and part 2 ‘Healthcare quality for professionals’). Each part includes 15 quality items and three general items.

### Multi-centre testing: sample

In total, 13,615 respondents (N_Patients/kin_ = 5,891 and N_Professionals_ = 7,724) completed the FlaQuM-Quickscan. The respondents’ characteristics are shown in Table 1. Among patients and kin, 56.4% were female and 32.9% were aged 51–65. Among professionals, 40.8% were nurses, 75.3% were female, and 48.2% were aged 31–50.

**Table 1:** Characteristics of respondents

	**Patients and kin Total (*N* = 5,891)**	**Professionals Total (*N* = 7,724)**
**Type of respondent, *N (%)***		
Patients/kin		
Patients	4,720 (80.1%)	/
Kin	1,171 (19.9%)	/
Professionals		
Management and boards	/	145 (1.9%)
Middle management (Staff members and supervisors)	/	898 (11.6%)
Physicians / Dentists	/	882 (11.4%)
Nurses / Midwives / Nursing assistants	/	3,152 (40.8%)
Other professionals with direct patient contact	/	1,531 (19.8%)
Supporting professionals without direct patient contact	/	1,036 (13.4%)
Professional group unknown	/	80 (1.0%)
**Gender, *N (%)***		
Female	3,322 (56.4%)	5,818 (75.3%)
Male	2,458 (41.7%)	1,820 (23.6%)
Other	11 (0.2%)	50 (0.6%)
Unknown	100 (1.7%)	36 (0.5%)
**Age (years), *N (%)***		
18–30	533 (9.1%)	1,453 (18.8%)
31–50	1,578 (26.8%)	3,723 (48.2%)
51–65	1,938 (32.9%)	2,449 (31.7%)
66–79	1,516 (25.7%)	55 (0.7%)
80 +	268 (4.6%)	8 (0.1%)
Unknown	58 (1.0%)	36 (0.5%)

### Descriptive results

Descriptive results of 15 items of the multidimensional quality model and the three general items are analysed for part 1 ‘Healthcare quality for patients and kin’ and part 2 ‘Healthcare quality for professionals’ [see Additional file 2]. For part 1, averages of items varied between 7.7 (‘Kin-centred care’ and ‘Eco-friendly’) and 8.7 (‘Equity’ and ‘Kindness with compassion’) and between 5.9 (‘Eco-friendly’) and 8.3 (‘Equity’) scored by patients/kin and professionals, respectively. The item with the lowest average was the same as the one with the highest percentage of scores between 0–5 (‘Eco-friendly’ scored by patients/kin and professionals) and vice versa for the highest percentage of scores between 8–10. For part 2, averages of items varied between 7.6 (‘Kin-centred care’, ‘Resilience’, ‘Partnership and co-production’) and 8.3 (‘Kindness with compassion’) and between 5.8 (‘Resilience’, ‘Efficient’, ‘Accessible and timely’ and ‘Partnership and co-production’) and 8.0 (‘Equity’) scored by patients/kin and professionals, respectively. The items with the lowest average were the same as the ones with the highest percentage of scores between 0–5 (‘Partnership and co-production’ scored by patients and kin, ‘Accessible and timely’ and ‘Partnership and co-production’ scored by professionals) and vice versa for the highest percentage of scores between 8–10. For all items, percentage distributions of scores for each item and averages on items scored by patients and kin were significantly different from those scored by professionals, except for the general item ‘Intention-to-stay’ in instrument part 2.

### Construct validity

The hypothesised dimensionality of part 1 ‘Healthcare quality for patients and kin’ and part 2 ‘Healthcare quality for professionals’ were evaluated separately (Table 2). The hypothesised subscales of both instrument parts were conceptualized as such by patients, kin and professionals. Moreover, the ICM-CFA and the multiple group ICM-CFA showed good to very good fit for the data for the respondent groups in both instrument parts [see Additional file 3]. The FlaQuM-Quickscan allowed for cross-group comparison between patients, their kin and professionals.

**Table 2:** Confirmatory factor analyses

	ɣ^2^	*p*	df	CFI	TLI	RMSEA (90% CI)
**Part 1 ‘Healthcare quality for patients and kin’**						
CFA (with 4 factors): Patients/kin	3558.069	< 0.001	84	0.961	0.951	0.084 (0.081—0.086)
CFA (with 4 factors): Professionals	4245.651	< 0.001	84	0.950	0.938	0.080 (0.078—0.082)
**Part 2 ‘Healthcare quality for professionals’**						
CFA (with 4 factors): Patients/kin	3667.058	< 0.001	84	0.969	0.962	0.085 (0.083—0.087)
CFA (with 4 factors): Professionals	5486.786	< 0.001	84	0.946	0.932	0.091 (0.089—0.093)

*CFI* Comparative fit index, *TLI *Tucker-Lewis index, *RMSEA* Root mean square error of approximation, *CFA* Confirmatory Factor Analysis

### Criterion validity

All correlation coefficients exceeded the 0.3 criterion. For part 1, associations of items-to-overall-quality-score ranged from 0.545 to 0.802 and from 0.373 to 0.713 responded by patients/kin and professionals respectively (Table 3). Associations of items-to-recommendation-score ranged from 0.494 to 0.790 and from 0.326 to 0.671 responded by patients/kin and professionals respectively. Associations of items-to-intention-to-stay-score ranged from 0.468 to 0.759 and from 0.309 to 0.608 responded by patients/kin and professionals respectively. The association of the item ‘Eco-friendly’ and the three general items of both parts, responded by patients/kin as well as professionals, is assessed as the lowest, except for the item ‘Equity’ responded by professionals in part 2. The association of the item ‘Dignity and respect’ and each general item of both parts and responded by patients/kin and professionals is considered the highest. For part 2, associations of items-to-overall-quality-score ranged from 0.697 to 0.812 and from 0.438 to 0.822 responded by patients/kin and professionals respectively. Associations of items-to-recommendation-score ranged from 0.654 to 0.777 (scored by patients/kin) and from 0.434 to 0.781 (scored by professionals). Associations of items-to-intention-to-stay-score ranged from 0.633 to 0.729 (scored by patients/kin) and from 0.417 to 0.637 (scored by professionals).

**Table 3:** Item-to-general-items correlations

	**Overall quality score**	**Recommendation score**	**Intention-to-stay score**
**Part 1 ‘Healthcare quality for patients and kin’**			
**Respondents: patients/kin**			
Person-centred	0.762	0.729	0.704
Kin-centred	0.689	0.660	0.629
Transparency	0.715	0.689	0.658
Leadership	0.771	0.742	0.714
Resilience	0.769	0.743	0.715
Safe	0.727	0.695	0.675
Effective	0.769	0.745	0.719
Efficient	0.723	0.683	0.658
Accessible and timely	0.727	0.694	0.669
Equity	0.643	0.637	0.618
Eco-friendly	0.545	0.494	0.468
Dignity and respect	0.802	0.790	0.759
Holistic	0.776	0.755	0.724
Partnership and co-production	0.778	0.745	0.714
Kindness with compassion	0.778	0.773	0.747
**Respondents: professionals**			
Person-centred	0.674	0.618	0.541
Kin-centred	0.639	0.572	0.502
Transparency	0.614	0.541	0.485
Leadership	0.631	0.567	0.506
Resilience	0.648	0.585	0.515
Safe	0.667	0.621	0.570
Effective	0.627	0.574	0.532
Efficient	0.581	0.523	0.479
Accessible and timely	0.577	0.511	0.462
Equity	0.490	0.481	0.439
Eco-friendly	0.373	0.326	0.309
Dignity and respect	0.713	0.671	0.608
Holistic	0.690	0.639	0.571
Partnership and co-production	0.663	0.602	0.539
Kindness with compassion	0.677	0.622	0.558
**Part 2 ‘Healthcare quality for professionals’**			
**Respondents: patients/kin**			
Person-centred	0.784	0.752	0.715
Kin-centred	0.776	0.739	0.706
Transparency	0.778	0.749	0.716
Leadership	0.774	0.753	0.704
Resilience	0.793	0.751	0.724
Safe	0.781	0.739	0.701
Effective	0.760	0.738	0.681
Efficient	0.776	0.739	0.700
Accessible and timely	0.757	0.732	0.677
Equity	0.698	0.675	0.633
Eco-friendly	0.697	0.654	0.633
Dignity and respect	0.812	0.777	0.729
Holistic	0.792	0.751	0.723
Partnership and co-production	0.791	0.751	0.718
Kindness with compassion	0.758	0.740	0.672
**Respondents: professionals**			
Person-centred	0.794	0.751	0.609
Kin-centred	0.724	0.683	0.563
Transparency	0.691	0.653	0.532
Leadership	0.669	0.653	0.536
Resilience	0.760	0.716	0.574
Safe	0.701	0.667	0.539
Effective	0.658	0.638	0.529
Efficient	0.673	0.628	0.495
Accessible and timely	0.694	0.651	0.510
Equity	0.438	0.442	0.417
Eco-friendly	0.467	0.434	0.350
Dignity and respect	0.822	0.781	0.637
Holistic	0.803	0.766	0.619
Partnership and co-production	0.756	0.718	0.557
Kindness with compassion	0.584	0.579	0.496

### Reliability

For part 1, the Cronbach’s alphas were 0.967 and 0.957 scored by patients/kin and professionals, respectively. The Cronbach’s alphas for the subscales were ranging from 0.828 to 0.937 (Table 4). For part 2, the Cronbach’s alphas were 0.981 and 0.947 scored by patients/kin and professionals, respectively. The Cronbach’s alphas for the subscales were ranging from 0.857 to 0.945.

**Table 4:** Internal consistency

Subscales	Patients’/kin’s Cronbach’s alphas	Professionals Cronbach’s alphas
**Part 1 ‘Healthcare quality for patients and kin’**		
Person- and kin-centred care	0.847	0.885
Catalysts	0.905	0.841
Technical domains	0.908	0.828
Core values	0.937	0.913
**Part 2 ‘Healthcare quality for professionals’**		
Person- and kin-centred care	0.930	0.892
Catalysts	0.937	0.861
Technical domains	0.945	0.857
Core values	0.945	0.897

### Process evaluation

In three pilot hospitals, presentations at committees, leaflets, paper posters and screensavers in waiting rooms were used to communicate about the FlaQuM-Quickscan. Healthcare quality managers, job students and volunteers were actively distributing the FlaQuM-Quickscan with a QR-code and tablets on which respondents could immediately complete it. In these hospitals, an individualised approach to explain FlaQuM-Quickscan objectives, to describe the added value of both instrument parts and to support in the online navigation was observed to be most effective. Based on the analysis of the researchers’ observation notes, a clear introduction and instructions on how to complete this mirror instrument emerged as essential. In part 1 of the FlaQuM-Quickscan, professionals without experience as a patient in that hospital were asked to imagine what it would be like to be a patient there. In part 2 of the FlaQuM-Quickscan, patients and kin that were not employed in that hospital, were asked to score the items based on what they could feel, hear and experience during their hospital contact. These lessons learned were presented to healthcare quality managers of included hospitals before the FlaQuM-Quickscan distribution was launched in their hospital. Interviews with 17 healthcare quality managers revealed that all hospitals explained FlaQuM-Quickscan objectives and added value on meetings with employees and used a personalised poster in the hospital’s language, leaflet or screensaver to communicate to all healthcare stakeholders, including patients and kin.

Six hospitals published an article in their hospital magazine and three hospitals launched an introduction video. For distribution, social media or internal webpages were used by three hospitals towards patients and kin and by nine hospitals towards professionals. Moreover, all hospitals used e-mail addresses of professionals to contact them and one hospital used text messages to reach patients. Additionally, to motivate patients, kin and professionals, all hospitals used an individualised approach with a job student or volunteer motivating respondents hospital-wide to complete the FlaQuM-Quickscan. In eleven hospitals they used tablets for immediate instrument completion. Moreover, hospitals received weekly feedback about the number of respondents for each type of respondent, which motivated them to focus on reaching lower response groups.

## Discussion

This study described a multi-step approach to develop and validate an instrument that measures experiences of QoC multidimensionally [2] from an integrated multistakeholder perspective, i.e. patients, kin and professionals. The goal of this mirror instrument is to measure patients’, kin’s and professionals’ experiences of quality in terms of care for patients and their kin (instrument part 1) and for professionals (instrument part 2). The FlaQuM-Quickscan is the first to provide a comprehensive, non-disease-specific assessment of QoC for both patients/kin and professionals. A mirror instrument has been used extensively in health services research to study different perspectives, e.g. to mirror experiences of different stakeholder groups, such as patients and professionals [17, 57, 58], or to mirror experiences of one stakeholder group focusing on different care perspectives [43]. The uniqueness of the FlaQuM-Quickscan is that all stakeholders complete both instrument parts, which implicates that patients and kin have to imagine how the hospital cares for professionals and vice versa. Mirroring experiences is substantially supported by theoretical models [2, 59] describing that experiential knowledge of patients and kin may differ from the gaps experienced and preferences held by professionals and vice versa. Integrating different perspectives gives the opportunity to analyse discrepancies and to foster an in-depth discussion to gain a deeper understanding on QoC [59]. The complementarity of quantitative and qualitative results to define QoC priorities, reinforce an integrated, well-informed approach towards quality management.

The validation of the FlaQuM-Quickscan started by conducting focus groups [35] and involving an expert advisory panel to establish content validity, followed by obtaining face validity through a pilot test in a multistakeholder group. Subsequent validation steps focused on a series of factor analytic models assessing multidimensionality and measurement invariance. The hypotheses to divide each instrument part in four subscales, as a priori defined in Lachman’s model, were confirmed in our multicentre study. This dimensionality fitted our stakeholder groups of patients/kin and professionals separately. Multiple group analyses showed a well-fitting model for both groups and allowed comparison across various types of respondents and their characteristics (gender and age). We assumed that respondents can only score on domains experienced by themselves, but based on validity tests we can conclude that items of each instrument part separately had the same meaning for each type of respondent. The criterion validity tests revealed that the majority of items demonstrated strong correlations with overall quality assessment of respondents, thus appearing to measure QoC and nothing else. Consistent with other research [60], the core value ‘Dignity and respect’ showed the highest correlation with the overall quality assessment in both instrument parts and for both stakeholder groups (patients/kin and professionals). Therefore, despite the generally accepted measurement of technical quality aspects, from a patients’, kin’s and professionals’ view the emphasis has to be on interpersonal, relational, interprofessional and behavioural aspects in quality management [12, 26, 31, 32]. The Cronbach’s alpha coefficients revealed good internal consistency for both instrument parts. These values are excellent in comparison with earlier studies that demonstrated lower range rates for instruments measuring healthcare quality experiences of patients [19, 22], kin [23] and professionals [12, 24, 26]. In conclusion, this validated instrument can facilitate co-production of a sustainable, multidimensional quality management system in which all stakeholders’ values are central.

Our process evaluation emphasised the need for an individualised approach in communicating and distributing the FlaQuM-Quickscan and in motivating stakeholders to share their QoC experiences. Although the domain ‘Eco-friendly’ is a maturing quality attribute receiving growing research attention [4], it correlates the lowest of all quality domains in our study. In the current paradigm of youth awareness for environmental conditions and climate targets, the domain may be correlated differently by younger respondents in our sample. Moreover, despite including health equity in the Quintuple Aim [13], the domain has the second lowest association with overall quality assessment. This may be due to the inclusion of only Dutch-speaking respondents in our sample. The FlaQuM-Quickscan can be expanded to include information on cultural backgrounds and socio-economic demographics of respondents.

FlaQuM-Quickscan results at meso- or micro-level can be used by hospitals to build a shared quality vision and to define related aims (FlaQuM pillar 1). In practice, the discrepancies between the experiences of patients, kin and professionals as well as the differences between results of FlaQuM-Quickscan part 1 ‘Healthcare quality for patients and kin’ and FlaQuM-Quickscan part 2 ‘Healthcare quality for professionals’ can be used for this vision development. The brief tool can be used to develop a monitoring and transparent feedback system, as guided in the co-creation roadmaps towards sustainable QoC (FlaQuM pillar 2) [14]. As shown in our study, monitoring quality multidimensionally implies a focus on technical experiences and soft skills. Education programmes are increasingly focusing on soft skills such as leadership and teamwork as important factors contributing to quality improvement [25, 61]. Hospital human resources departments can use FlaQuM-Quickscan results to improve patient, kin and employer experience [13]. Moreover, the FlaQuM-Quickscan could be expanded to include items concerning care pathways, protocols or procedures as well as the quality of communication between patient and provider. In addition to in-hospital QoC management, benchmark reports can be shared to learn during inter-hospital learning collaboratives (FlaQuM pillar 3). In conclusion, the FlaQuM-Quickscan will be useful to researchers, healthcare managers, hospitals’ executives and policymakers. In future research, variation in experiences within and between stakeholder groups and hospitals can be examined to identify quality priorities at management, Executive and Board levels and to co-produce future quality initiatives. Additionally, associations of experiences and respondents’ demographic variables will be researched. When data from repeated measurements become available, longitudinal invariance and impact of quality initiatives on FlaQuM-Quickscan scores must be studied to explore the sensitivity of the instrument.

### Strengths and limitations

A major strength of this study is the evidence-based, stepwise development of this new instrument in a multi-centre setting of 17 hospitals and a parallel process evaluation. The sample of patients, kin and professionals consisted of a female/male ratio that is similar to other healthcare studies [18, 62]. Inclusion criteria were only restricted by age, which might lead to a generalisability of results in hospital settings. Quality is addressed multidimensionally in each instrument part, which are validated separately and can be used to mirror results of both parts and of both perspectives. Subscales or individual quality domains can be used on their own. Because the validation of this multidimensional instrument is complex, with analyses per respondent group and per FlaQuM-Quickscan part, and because previous studies used different types of analyses based on the characteristics of their developed instrument, this study did not make a statement on the comparison of our validation results with those of other instruments. The approach of the FlaQuM-Quickscan is efficient (not time-consuming), feasible and therefore useful for formal quality improvement methods that put patients’, kin’s and professionals’ experiences central. Although this instrument has been developed in Flanders, the method of the FlaQuM-Quickscan could be applied in all healthcare settings in an international perspective. Potential limitations of this study are the cross-sectional design and the self-administrating instrument completion. Further testing of psychometric properties, such as content validity index and convergent validity, is preferable. Evaluation of the FlaQuM-Quickscan in other languages, different countries and in the wider context of healthcare systems, such as in primary care settings, will be the focus of future research. Additionally, within the methods of this study we were not able to match patients, kin and professionals around individual patient cases. Future studies should focus on matched analysis and on understanding differences between experiences of the different stakeholders.

## Conclusions

Based on a multi-method approach to establish content and face validity followed by the assessment of construct validity, criterion validity as well as the reliability, the FlaQuM-Quickscan is considered as valid to measure and mirror experiences of QoC multidimensionally from a multistakeholder perspective, i.e. patients, kin and professionals. The FlaQuM-Quickscan measures ‘Healthcare quality for patients and kin’ (part 1) and ‘Healthcare quality for professionals’ (part 2). Each instrument part contains 15 quality items, reflecting quality domains, and 3 general items. The power of this new instrument is its ability to mirror experiences from patients, kin and professionals, providing unique and detailed data to design a sustainable quality management system in hospitals. Continuous monitoring of stakeholders’ experiences can serve as a catalyst for quality improvement. Future research will assess the transferability to other healthcare systems, examine between-stakeholder group and between-hospitals variation and support to set national quality priorities.

### Supplementary Information

**Additional file 1. **FlaQuM-Quickscan survey

**Additional file 2. **Descriptive results

**Additional file 3. **Good-of-fit indices

## Data Availability

The datasets generated during and analysed during the current study are not publicly available due to containing information that could compromise the privacy of research participants but are available from the corresponding author on reasonable request and with permission of all local ethics committees of participating hospitals.

## Abbreviations

IOM: Institute of Medicine
CFA: Confirmatory factor analysis
RITA: Rapid identification of themes from audio recordings
QoC: Quality of care
FlaQuM: Flanders Quality Model
CFI: Comparative fit index
ICM-CFA: Independent cluster model confirmatory factor analysis
RMSEA: Root mean square error of approximation
TLI: Tucker-Lewis index

## References

[1] Institute of Medicine (US) Committee on Quality of Health Care America (2001). Crossing the Quality Chasm.

[2] Lachman P, Batalden P, Vanhaecht K (2021). A multidimensional quality model: an opportunity for patients, their kin, healthcare providers and professionals to coproduce health. F1000Res.

[3] Vanhaecht K, De Ridder D, Seys D, Brouwers J, Claessens F, Van Wilder A (2021). The History of Quality: From an Eye for an Eye, Through Love, and Towards a Multidimensional Concept for Patients, Kin, and Professionals. Eur Urol Focus.

[4] Sherman JD, Thiel C, MacNeill A, Eckelman MJ, Dubrow R, Hopf H (2020). The Green Print: Advancement of Environmental Sustainability in Healthcare. Resour Conserv Recycl..

[5] DesRoches CM (2020). Healthcare in the new age of transparency. Semin Dial.

[6] Black P, Boore JRP, Parahoo K (2011). The effect of nurse-facilitated family participation in the psychological care of the critically ill patient. J Adv Nurs.

[7] Mackie BR, Mitchell M, Marshall PA (2018). The impact of interventions that promote family involvement in care on adult acute-care wards: An integrative review. Collegian.

[8] Berger Z, Flickinger TE, Pfoh E, Martinez KA, Dy SM, Berger ZD. Promoting engagement by patients and families to reduce adverse events in acute care settings: a systematic review. 10.1136/bmjqs. 10.1136/bmjqs-2012-001769 PMC4079036 24336575

[9] Goodwin N, Brown A, Johnson H, Miller R, Stein KV (2022). From People-Centred to People-Driven Care: Can Integrated Care Achieve its Promise without it?. Int J Integr Care..

[10] Garcia CDL, de Abreu LC, Ramos JLS, de Castro CFD, Smiderle FRN, dos Santos JA (2019). Influence of burnout on patient safety: systematic review and meta-analysis. Medicina (Lithuania)..

[11] Tawfik DS, Scheid A, Profit J, Shanafelt T, Trockel M, Adair KC (2019). Evidence relating health care provider burnout and quality of care a systematic review and meta-analysis. Ann Intern Med.

[12] Maassen SM, Weggelaar Jansen AMJW, Brekelmans G, Vermeulen H, Van Oostveen CJ (2020). Psychometric evaluation of instruments measuring the work environment of healthcare professionals in hospitals: A systematic literature review. Int J Qual Health Care.

[13] Nundy S, Cooper LA, Mate KS (2022). The Quintuple Aim for Health Care Improvement: A New Imperative to Advance Health Equity. JAMA - Journal of the American Medical Association.

[14] Claessens F, Seys D, Brouwers J, Van Wilder A, Jans A, Castro EM (2022). A co-creation roadmap towards sustainable quality of care: A multi-method study. PLoS ONE.

[15] Batalden P, Foster T (2021). From assurance to coproduction: A century of improving the quality of health-care service. Int J Qual Health Care.

[16] Stavropoulou A, Rovithis M, Kelesi M, Vasilopoulos G, Sigala E, Papageorgiou D (2022). What Quality of Care Means? Exploring Clinical Nurses’ Perceptions on the Concept of Quality Care: A Qualitative Study. Clin Pract.

[17] Woods EJ, Ginsburg AD, Bellolio F, Walker LE (2020). Palliative care in the emergency department: A survey assessment of patient and provider perspectives. Palliat Med.

[18] Hesselink G, Schoonhoven L, Plas M, Wollersheim H, Vernooij-Dassen M (2013). Quality and safety of hospital discharge: A study on experiences and perceptions of patients, relatives and care providers. Int J Qual Health Care.

[19] Zinckernagel L, Schneekloth N, Zwisler ADO, Ersbøll AK, Rod MH, Jensen PD (2017). How to measure experiences of healthcare quality in Denmark among patients with heart disease? The development and psychometric evaluation of a patient-reported instrument. BMJ Open..

[20] Beattie M, Murphy DJ, Atherton I, Lauder W (2015). Instruments to measure patient experience of healthcare quality in hospitals: a systematic review. Syst Rev.

[21] European Patients Forum (2017). “Patients’’ Perceptions of Quality in Healthcare".”.

[22] Brown SM, McBride G, Collingridge DS, Butler JM, Kuttler KG, Hirshberg EL (2015). Validation of the Intermountain patient perception of quality (PPQ) survey among survivors of an intensive care unit admission: A retrospective validation study Quality, performance, safety and outcomes. BMC Health Serv Res..

[23] Van Mol MMC, Bakker EC, Nijkamp MD, Kompanje EJO, Bakker J, Verharen L (2014). Relatives’ perspectives on the quality of care in an Intensive Care Unit: The theoretical concept of a new tool. Patient Educ Couns.

[24] Kamalasanan A, Sathiyamurthi G, Subbarayalu AV (2020). A tool to assess the quality perception of healthcare employees. Int J Health Care Qual Assur.

[25] Gauld R, Horsburgh S (2014). Healthcare professional perspectives on quality and safety in New Zealand public hospitals: Findings from a national survey. Aust Health Rev.

[26] Harrison R, Manias E, Ellis L, Mimmo L, Walpola R, Roxas-Harris B (2022). Evaluating clinician experience in value-based health care: the development and validation of the Clinician Experience Measure (CEM). BMC Health Serv Res..

[27] Price RA, Elliott MN, Zaslavsky AM, Hays RD, Lehrman WG, Rybowski L (2014). Examining the role of patient experience surveys in measuring health care quality. Med Care Res Rev.

[28] Seppala EM, Hutcherson CA, Nguyen DT, Doty JR, Gross JJ. Loving-kindness meditation: a tool to improve healthcare provider compassion, resilience, and patient care. J Compassionate Health Care. 2014;1:5. https://jcompassionatehc.biomedcentral.com/articles/10.1186/s40639-014-0005-9#citeas. 10.1186/s40639-014-0005-9.

[29] Mosadeghrad AM (2013). Healthcare service quality: Towards a broad definition. Int J Health Care Qual Assur.

[30] Firth-Cozens J, Mowbray D (2001). Leadership and the quality of care. Qual Health Care.

[31] Gelkop C, Kagan I, Rozani V (2022). Are emotional intelligence and compassion associated with nursing safety and quality care? A cross-sectional investigation in pediatric settings. J Pediatr Nurs.

[32] Feo R, Conroy T, Wiechula R, Rasmussen P, Kitson A (2020). Instruments measuring behavioural aspects of the nurse–patient relationship: A scoping review. J Clin Nurs.

[33] Boateng GO, Neilands TB, Frongillo EA, Melgar-Quiñonez HR, Young SL (2018). Best Practices for Developing and Validating Scales for Health, Social, and Behavioral Research: A Primer. Front Public Health..

[34] Badu E, O’Brien AP, Mitchell R (2022). Review of analysis techniques in mental health research with consumer instruments – a guide for researchers. Ment Health Rev J.

[35] Claessens F, Castro EM, Jans A, Jacobs L, Seys D, Van Wilder A (2022). Patients’ and kin’s perspective on healthcare quality compared to Lachman’s multidimensional quality model: Focus group interviews. Patient Educ Couns.

[36] Backman C, Cho-Young D (2019). Engaging patients and informal caregivers to improve safety and facilitate person- and family-centered care during transitions from hospital to home – a qualitative descriptive study. Patient Prefer Adherence.

[37] Everall AC, Guilcher SJT, Cadel L, Asif M, Li J, Kuluski K (2019). Patient and caregiver experience with delayed discharge from a hospital setting: A scoping review. Health Expect.

[38] Cambridge University Press (2013). Cambridge Dictionary.

[39] Giordano LA, Elliott MN, Goldstein E, Lehrman WG, Spencer PA (2010). Development, implementation, and public reporting of the HCAHPS survey. Med Care Res Rev.

[40] Bruyneel L, Van Houdt S, Coeckelberghs E, Sermeus W, Tambuyzer E, Cosemans P (2018). Patient experiences with care across various types of mental health care: Questionnaire development, measurement invariance, and patients’ reports. Int J Methods Psychiatr Res..

[41] Flanders Quality Model. https://flaqum.org/english/. Accessed 6 Dec 2022.

[42] Tsang S, Royse CF, Terkawi AS (2017). Guidelines for developing, translating, and validating a questionnaire in perioperative and pain medicine. Saudi J Anaesth.

[43] Suhonen R, Leino-Kilpi H, Välimäki M (2005). Development and psychometric properties of the Individualized Care Scale. J Eval Clin Pract.

[44] Steenkamp JBEM, Baumgartner H (1998). Assessing measurement invariance in cross-national consumer research. Journal of Consumer Research.

[45] Muthén LK, Muthén BO. Mplus User’s Guide. Eight Edition. Los Angeles: Muthén & Muthén; 1998-2017. https://www.statmodel.com/download/usersguide/MplusUserGuideVer_8.pdf.

[46] Hu L, Bentler PM (1999). Cutoff criteria for fit indexes in covariance structure analysis: Conventional criteria versus new alternatives. Struct Equ Modeling.

[47] Chen FF (2007). Sensitivity of Goodness of Fit Indexes to Lack of Measurement Invariance. Struct Equ Modeling.

[48] Tucker LR, Lewis C (1973). A reliability coefficient for maximum likelihood factor analysis. Psychometrika.

[49] Livote EE, Wyka KE (2009). Introduction to Structural Equation Modeling Using SPSS and AMOS. Struct Equ Modeling.

[50] Statistical CJ, Analysis P (1992). Curr Dir Psychol Sci.

[51] Aiken LH, Sermeus W, Van Den Heede K, Sloane DM, Busse R, McKee M (2012). Patient safety, satisfaction, and quality of hospital care: Cross sectional surveys of nurses and patients in 12 countries in Europe and the United States. BMJ (Online)..

[52] Herrera CN, Guirardello EDB (2023). Patient Safety Climate, Quality of Care, and Intention of Nursing Professionals to Remain in Their Job During the COVID-19 Pandemic. J Patient Saf.

[53] Busse R, Klazinga N, Panteli D, Quentin W (2019). Health Policy Series No. 53 The editors Improving healthcare quality in Europe Characteristics, effectiveness and implementation of different strategies.

[54] IDEWE. Risicoanalyse psychosociaal welzijn als onderdeel van een psychosociaal welzijnsbeleid. https://www.idewe.be/-/rapsi-zorg. 2022.

[55] Neal JW, Neal ZP, VanDyke E, Kornbluh M (2015). Expediting the Analysis of Qualitative Data in Evaluation: A Procedure for the Rapid Identification of Themes From Audio Recordings (RITA). Am J Eval.

[56] Pope C (2000). Qualitative research in health care: Analysing qualitative data. BMJ.

[57] Zlateva I, Anderson D, Coman E, Khatri K, Tian T, Fifield J (2015). Development and validation of the Medical Home Care Coordination Survey for assessing care coordination in the primary care setting from the patient and provider perspectives. BMC Health Serv Res.

[58] Ringwald A, Goetz K, Steinhaeuser J, Fleischmann N, Schüssler A, Flaegel K (2021). Measuring care coordination in German primary care – adaptation and psychometric properties of the Medical Home Care Coordination Survey. BMC Health Serv Res..

[59] Berland A (2017). Using the Johari Window to explore patient and provider perspectives. International Journal of Health Governance.

[60] Ali SH, Ndubisi NO (2011). The effects of respect and rapport on relationship quality perception of customers of small healthcare firms. Asia Pacific Journal of Marketing and Logistics..

[61] Goldman J, Wong BM (2020). Nothing soft about ‘soft skills’: core competencies in quality improvement and patient safety education and practice. BMJ Qual Saf.

[62] Boniol M, Mcisaac M, Xu L, Wuliji T, Diallo K, Campbell J (2019). Gender equity in the health workforce: Analysis of 104 countries.

