# Supplementary material for: Measuring in-hospital quality multidimensionally by integrating patients’, kin’s and healthcare professionals’ perspectives: development and validation of the FlaQuM-Quickscan
Source: BMC Health Serv Res. 2023 Dec 16;23:1426. doi: 10.1186/s12913-023-10349-2 (PMC10725024; doi:10.1186/s12913-023-10349-2)
Supplement: Supplementary file 1 — Additional file 1. FlaQuM-Quickscan survey [file 12913_2023_10349_MOESM1_ESM.docx]

*Additional file 1: FlaQuM-Quickscan survey*

The FlaQuM-Quickscan contains two parts. Patients, kin, and professionals were asked to complete both instrument parts. The first part explores perspectives on *Healthcare quality for patients and kin*, i.e., how professionals care for patients and their kin, while the second part focuses on *Healthcare quality for professionals*, i.e., how the organisation cares for their professionals.

Each item is rated on a 11-point Likert-type scale.

Responses to the first 15 instrument items on quality domains of healthcare will reflect the respondent’s level of disagreement or agreement with the item statement, scored from *0* (strongly disagree) to *10* (strongly agree).

The first general item includes the overall quality assessment of received care (in part 1) and the overall quality assessment of the hospital as employer (in part 2), and is scored from *0* (worst possible quality) to *10* (best possible quality). The second general item concerns the willingness to recommend the hospital to family and friends for receiving care (in part 1) or to work as an employee (in part 2), scored from *0* (definitely no) to *10* (definitely yes). The third general item reflects on a respondent’s intention-to-stay in the next year to receive care (in part 1) or to work as employee (in part 2), scored from *0* (definitely no) to *10* (definitely yes).

**Part 1: Healthcare quality for patients and kin**

| **Instrument items** | Quality domains |
| --- | --- |
| This organisation takes into account the wishes, needs and requirements of patients. | Person-centred |
| This organisation pays close attention to family, caregivers and/or other kin. | Kin-centred |
| Patients and kin are informed about the quality of care in this organisation. | Transparency |
| All staff consistently demonstrate their commitment to the organisation and set a good example so that patients and their kin feel comfortable and safe. | Leadership |
| This organisation takes into account what a patient and their kin can cope with (e.g. stress or new information). | Resilience |
| The care provided to patients and kin in this organisation is safe and actions are taken to prevent or resolve unsafe situations. | Safe |
| The staff in this organisation know their jobs and are adequately trained for it. | Effective |
| This organisation takes actions to avoid unnecessary or duplicate activities in care and reduces the administrative burden on patients where possible. | Efficient |
| Care services are always accessible and offered without postponement or unnecessary delay. | Accessible and timely |
| All patients and kin are welcome, without any discrimination, based on gender, ethnicity, financial situation, sexual orientation or disability. | Equity |
| This organisation has a policy to reduce its ecological footprint, for example by means of reducing plastic use, by sorting waste and by water and energy management. | Eco-friendly |
| This organisation treats patients and their kin with dignity and respect. | Dignity and respect |
| This organisation considers the individual behind the patient and their kin: physical, spiritual, emotional, social and mental health are important. | Holistic |
| In this organisation patients and their kin are involved in decisions, listened to and their knowledge and experience are taken into account. | Partnership and co-production |
| In this organisation people are friendly and kind to patients and their loved ones. | Kindness with compassion |
| Which score would you give the overall quality of care, provided to patients and their kin, in this organisation? | Overall quality score |
| Would you recommend this organisation to your friends and family? | Recommendation score |
| If you need care in the coming year, would you choose this care organisation? | Intention-to-stay score |

**Part 2: Healthcare quality for professionals**

| **Statements** | Domain |
| --- | --- |
| This organisation takes into account the wishes, needs and requirements of staff. | Person-centred care |
| This organisation takes into account familial circumstances of staff. | Kin-centred care |
| Staff are informed about the quality of care in this organisation. | Transparency |
| Staff always show their commitment and set a good example that makes other staff feel trusted and safe. | Leadership |
| In this organisation, what staff can cope with (e.g. stress or new information), is taken into account. | Resilience |
| This organisation does everything within their power to keep a safe working environment for its staff and ensures that they dare to call each other to account for unsafe situations. | Safe |
| This organisation ensures that staff know their job and are adequately trained for it. | Effective |
| This organisation takes actions to avoid unnecessary or duplicate activities in care and reduces the administrative burden on staff where possible. | Efficient |
| This organisation ensures an adequate staffing level that works together optimally to provide care that is accessible, timely and without unnecessary delays. | Accessible and timely |
| All staff are welcome, without any discrimination, based on gender, ethnicity, financial situation, sexual orientation or disability. | Equity |
| Staff is motivated to reduce their environmental footprint, for example by means of reducing plastic use, by sorting waste and by water and energy management. | Eco-friendly |
| This organisation treats staff with dignity and respect. | Dignity and respect |
| This organisation considers the individual behind the staff: physical, spiritual, emotional, social and mental health are important. | Holistic |
| In this organisation, staff is actively involved in decisions, changes or improvement projects; they are listened to and their knowledge and experience is taken into account. | Partnership and co-production |
| In this organisation staff are friendly and kind to each other. | Kindness with compassion |
| Which score would you give this organisation as an employer? | Overall quality score |
| Would you recommend this organisation as an employer to your friends and family? | Recommendation score |
| Would you continue to work in this organisation in the coming year? | Intention-to-stay score |
