# Supplementary material for: Measuring in-hospital quality multidimensionally by integrating patients’, kin’s and healthcare professionals’ perspectives: development and validation of the FlaQuM-Quickscan
Source: BMC Health Serv Res. 2023 Dec 16;23:1426. doi: 10.1186/s12913-023-10349-2 (PMC10725024; doi:10.1186/s12913-023-10349-2)
Supplement: Supplementary file 2 — Additional file 2. Descriptive results [file 12913_2023_10349_MOESM2_ESM.docx]

*Additional file 2: Descriptive results*


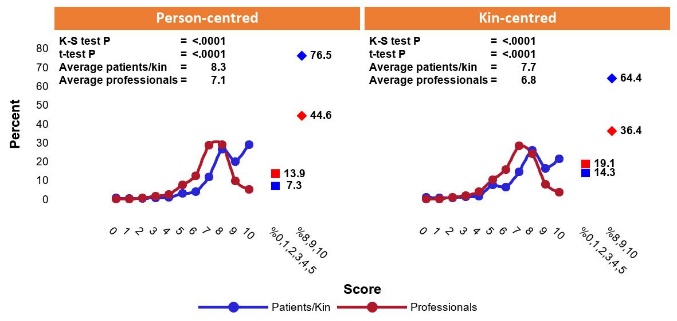

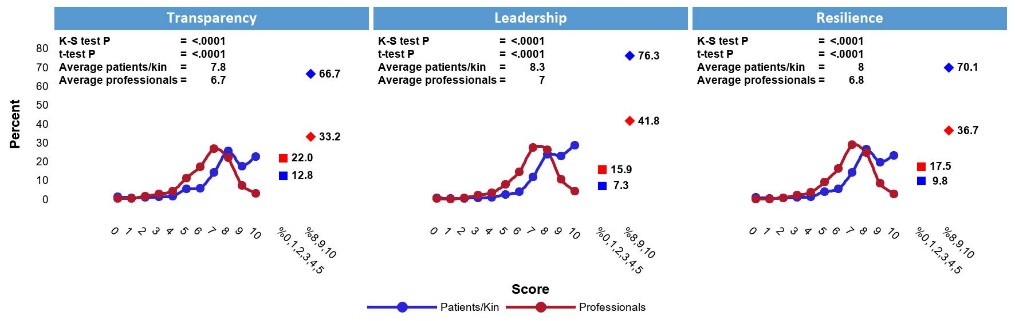

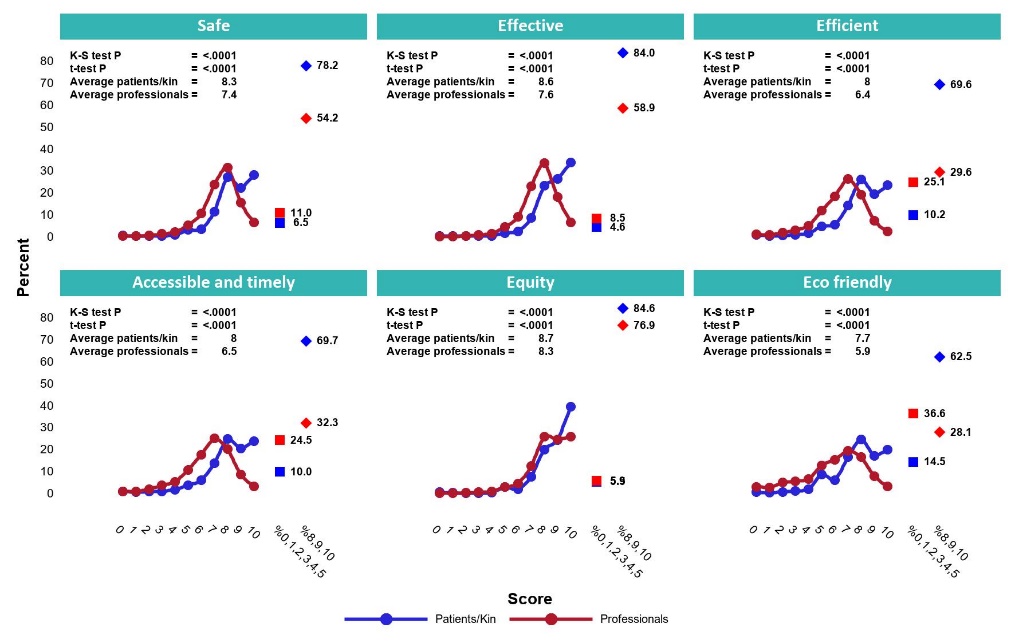

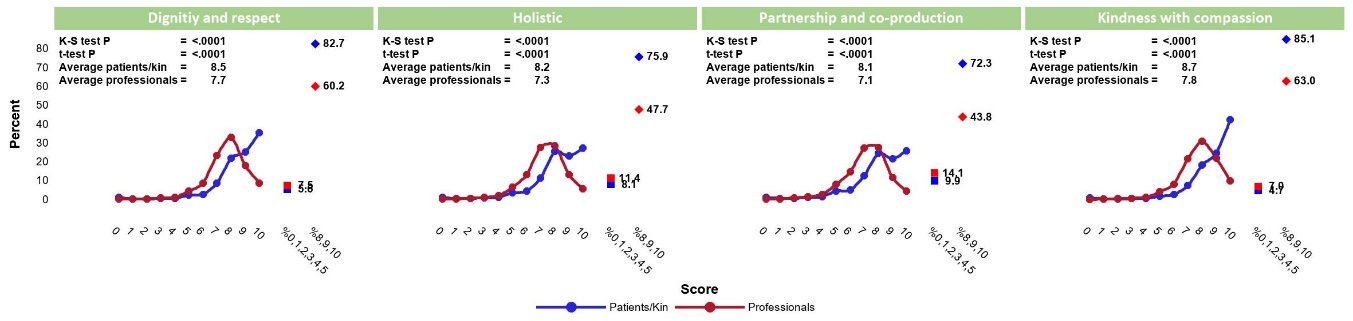

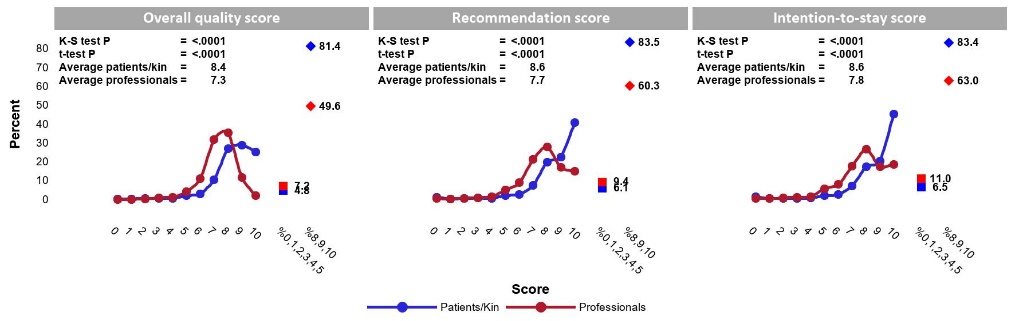


**Supplementary Fig. 1** Distribution of scores for part 1 ‘Healthcare quality for patients and kin’ on the 15 items reflecting quality domains (person- and kin-centred care: orange, catalysts: blue, technical domains: turquoise and core values: green) and 3 general items (grey) scored by patients/kin (dark blue line) and professionals (burgundy line). Next to the distribution of each item, the percentage of scores between 0-5 (square) and between 8-10 are shown (diamond). K-S test P = the p-value of the Kolmogorov-Smirnov test to assess differences in percentage distributions of scores between patients/kin and professionals. T-test p = the p-value of the t-test to assess differences in averages of items scored by patients/kin and by professionals.


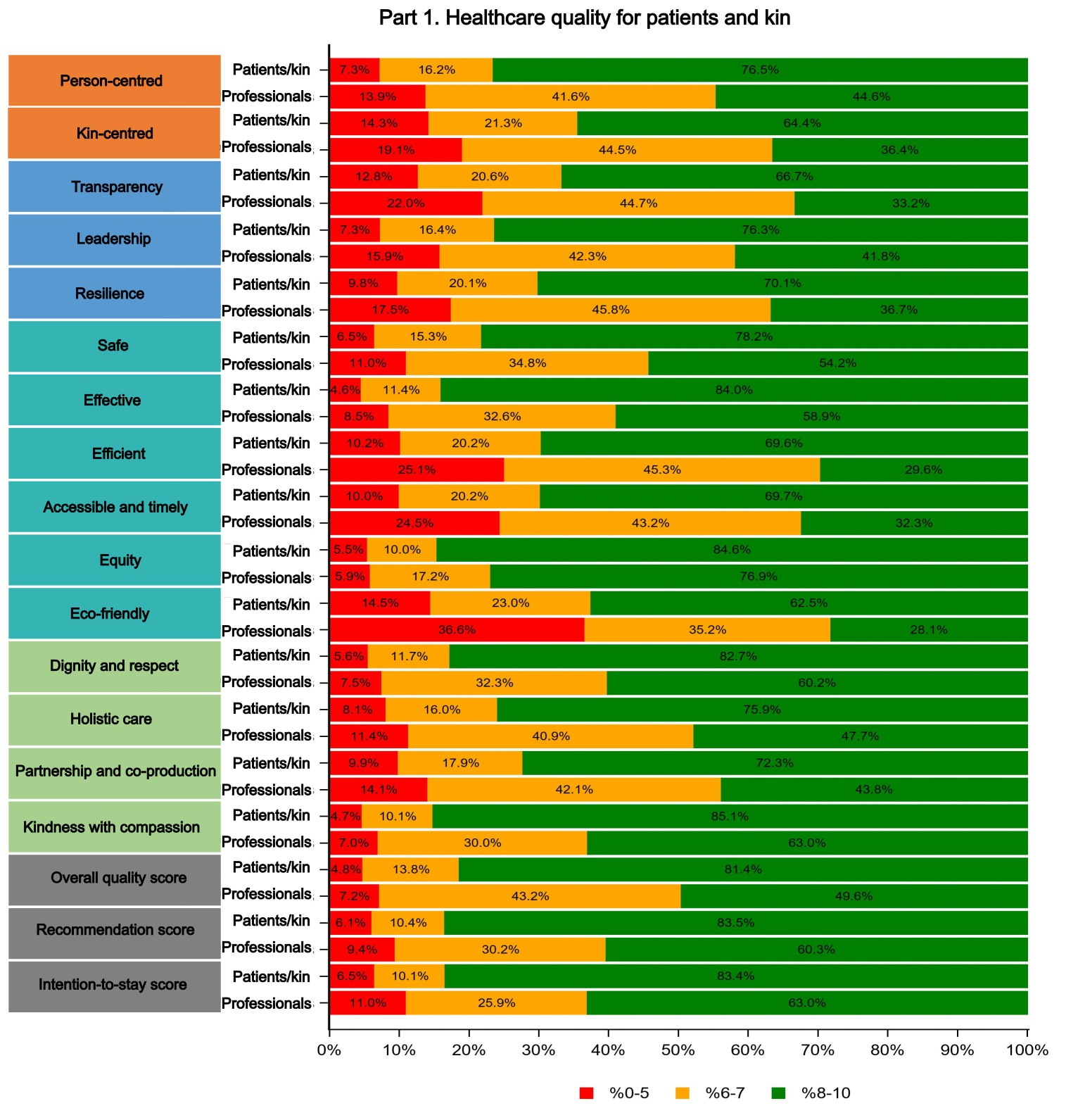


**Supplementary Fig. 2** The percentage of scores between 0-5 (red), between 6-7 (light orange) and between 8-10 (dark green) are shown for part 1 ‘Healthcare quality for patients and kin’ on the 15 items reflecting quality domains (person- and kin-centred care: orange, catalysts: blue, technical domains: turquoise and core values: green) and 3 general items (grey) scored by patients/kin (top bar for each quality domain) and professionals (bottom bar for each quality domain).


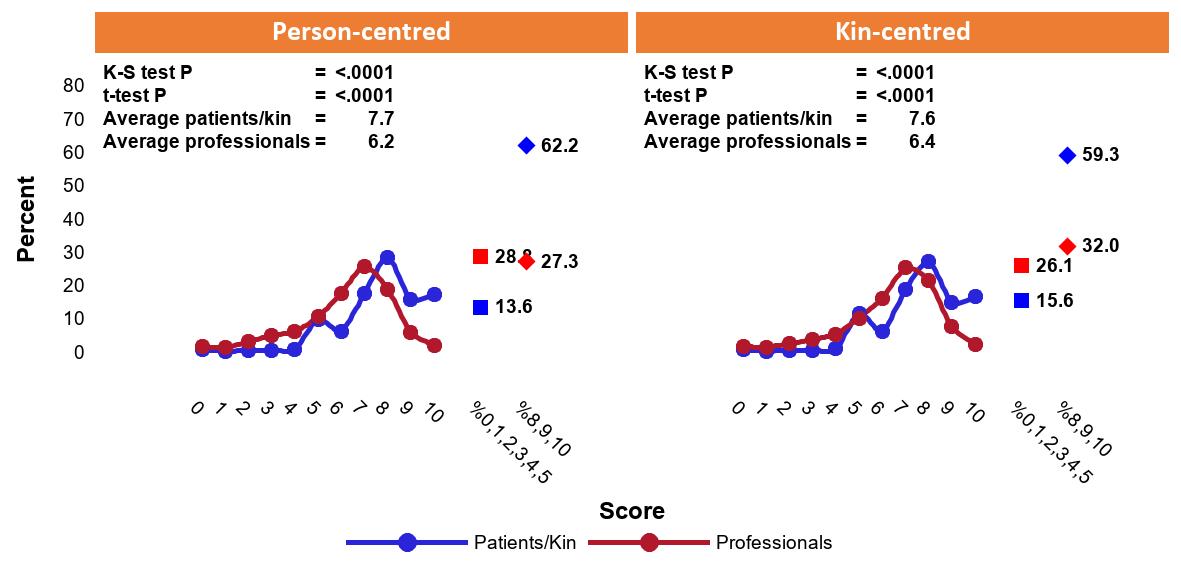

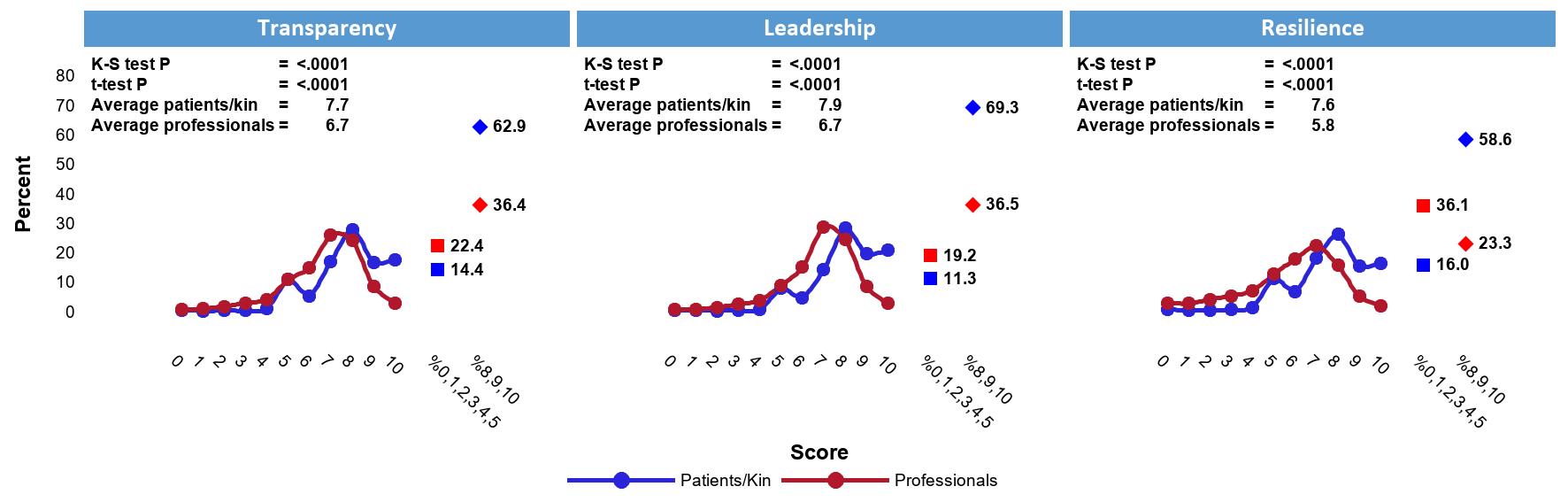

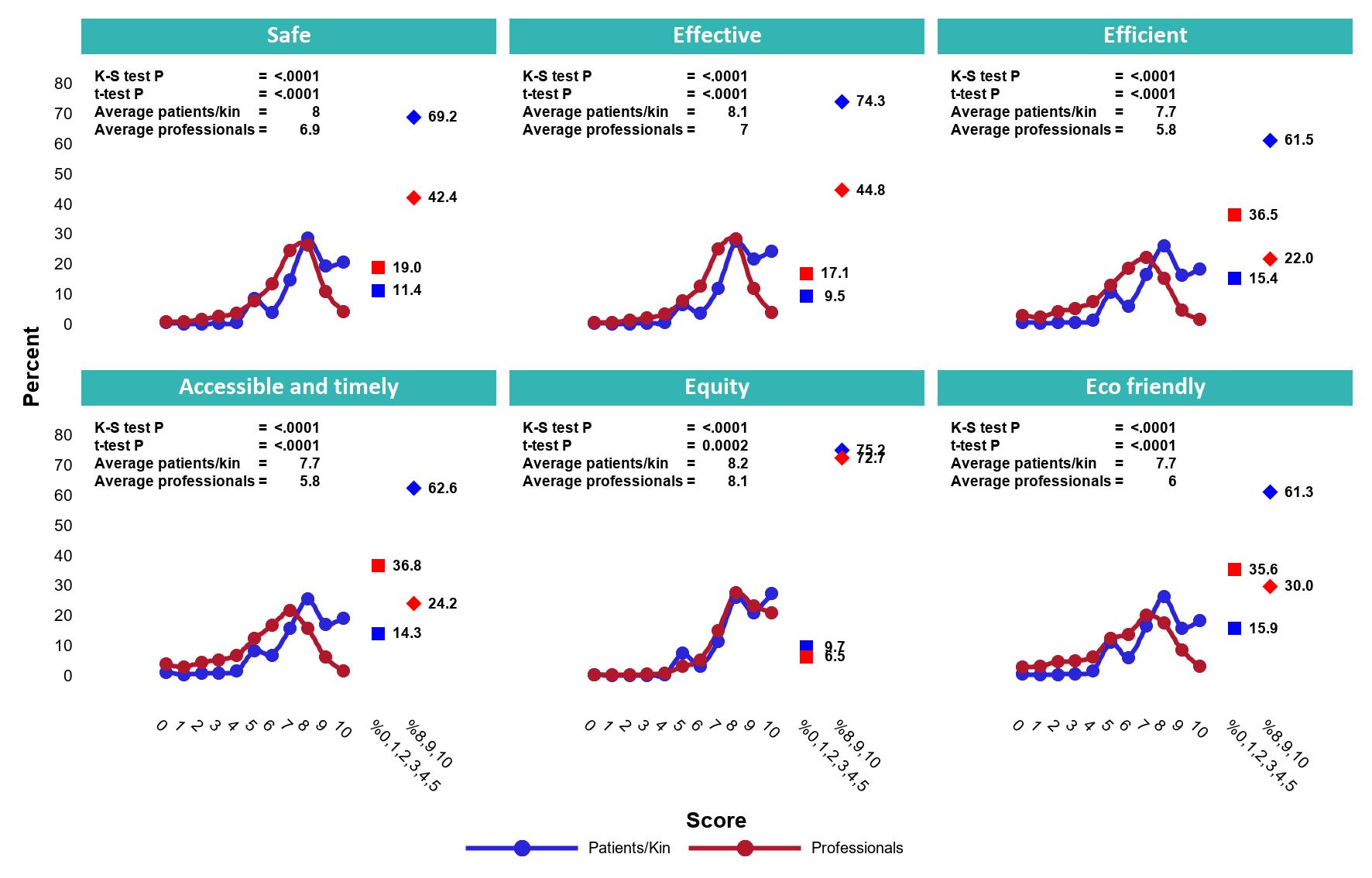

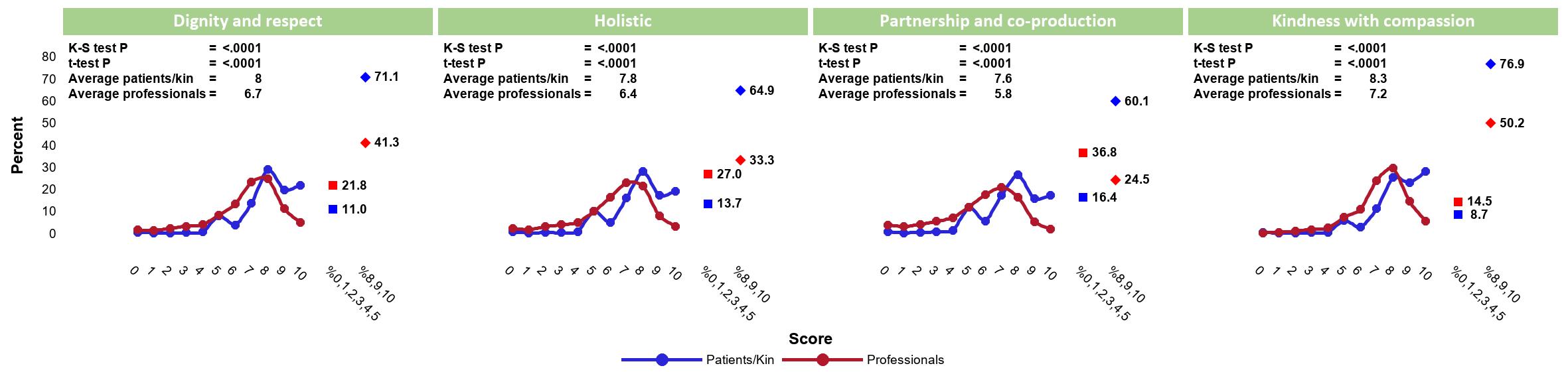

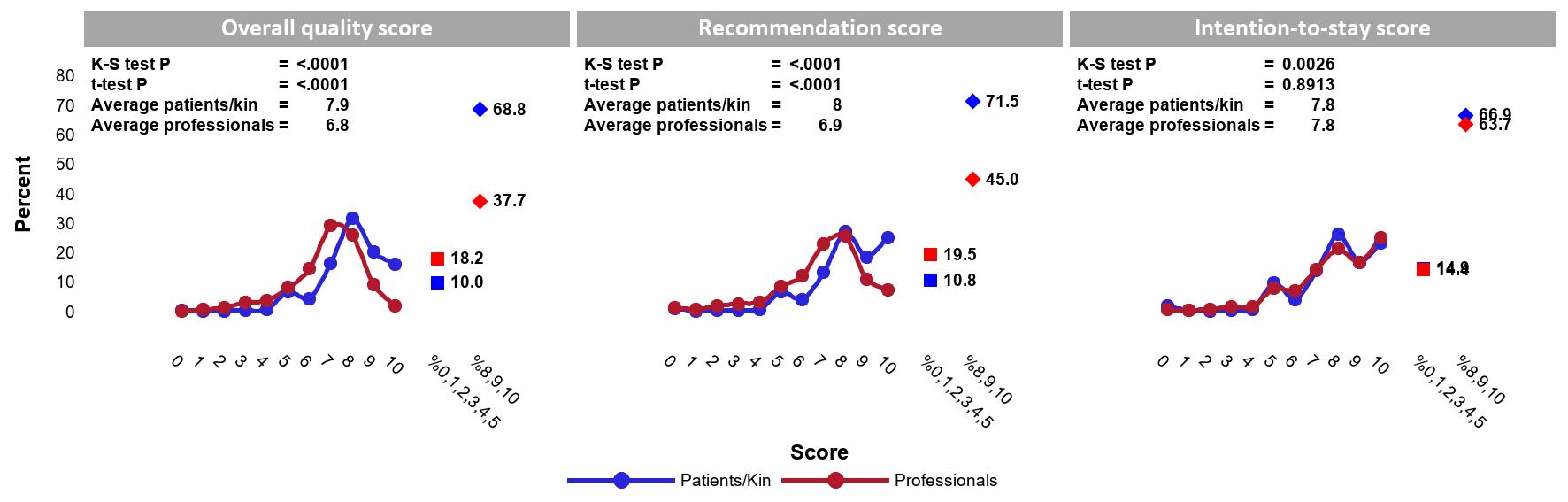


**Supplementary Fig. 3** Distribution of scores for part 2 ‘Healthcare quality for professionals’ on the 15 items of the multidimensional quality model (person- and kin-centred care: orange, catalysts: blue, technical domains: turquoise and core values: green) and 3 general items (grey) scored by patients/kin (dark blue line) and professionals (burgundy line). Next to the distribution of each item, the percentage of scores between 0-5 (square) and between 8-10 are shown (diamond). K-S test P = the p-value of the Kolmogorov-Smirnov test to assess differences in percentage distributions of scores between patients/kin and professionals. T-test p = the p-value of the t-test to assess differences in averages of items scored by patients/kin and by professionals.


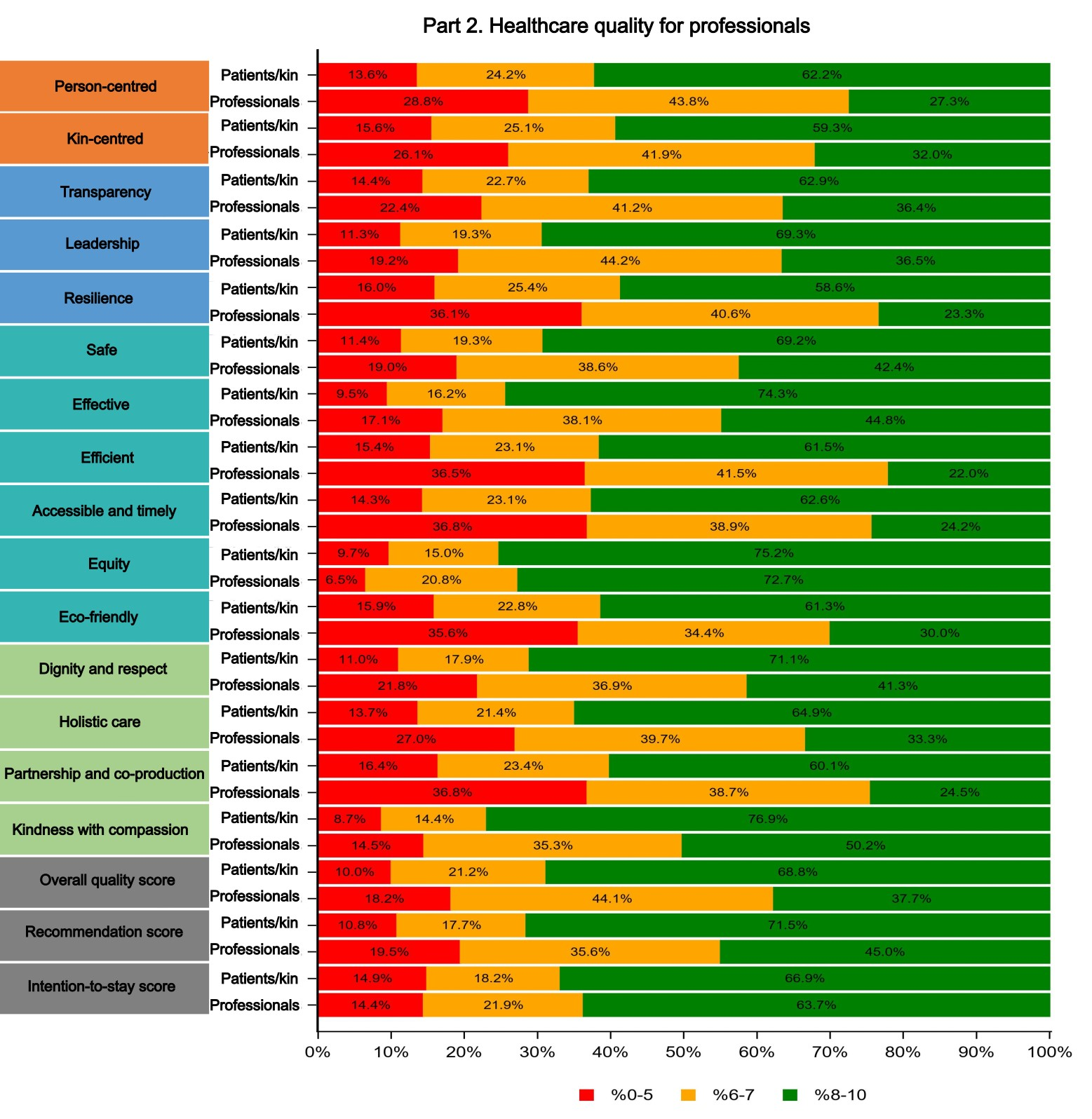


**Supplementary Fig. 4** The percentage of scores between 0-5 (red), between 6-7 (light orange) and between 8-10 (dark green) are shown for part 2 ‘Healthcare quality for professionals’ on the 15 items reflecting quality domains (person- and kin-centred care: orange, catalysts: blue, technical domains: turquoise and core values: green) and 3 general items (grey) scored by patients/kin (top bar for each quality domain) and professionals (bottom bar for each quality domain).
