# Supplementary material for: Measuring in-hospital quality multidimensionally by integrating patients’, kin’s and healthcare professionals’ perspectives: development and validation of the FlaQuM-Quickscan
Source: BMC Health Serv Res. 2023 Dec 16;23:1426. doi: 10.1186/s12913-023-10349-2 (PMC10725024; doi:10.1186/s12913-023-10349-2)
Supplement: Supplementary file 3 — Additional file 3. Good-of-fit indices [file 12913_2023_10349_MOESM3_ESM.docx]

*Additional file 3: Good-of-fit indices*

**Supplementary Table 1** Goodness-of-fit indices associated with factor analyses for part 1 ‘Healthcare quality for patients and kin’

|  | ɣ² | p | df | CFI | TLI | RMSEA (90% CI) |
| --- | --- | --- | --- | --- | --- | --- |
| **Respondents: Patients and kin** | | | | | | |
| Type of respondents | | | | | | |
| ICM-CFA with 4 factors: Patients | 2901.370 | <0.001 | 84 | 0.959 | 0.948 | 0.085 (0.082 - 0.087) |
| ICM-CFA with 4 factors: Kin | 858.220 | <0.001 | 84 | 0.960 | 0.950 | 0.089 (0.084 - 0.095) |
| Multiple group ICM-CFA with 4 factors: Configural invariance | 3759.589 | <0.001 | 168 | 0.959 | 0.949 | 0.086 (0.083 – 0.088) |
| Multiple group ICM-CFA with 4 factors: Scalar invariance | 3892.085 | <0.001 | 190 | 0.958 | 0.953 | 0.082 (0.080 - 0.084) |
| Gender | | | | | | |
| ICM-CFA with 4 factors: Female | 2336.923 | <0.001 | 84 | 0.956 | 0.945 | 0.090 (0.087 - 0.093) |
| ICM-CFA with 4 factors: Male | 1470.582 | <0.001 | 84 | 0.961 | 0.951 | 0.081 (.0078 - 0.085) |
| Multiple group ICM-CFA with 4 factors: Configural invariance | 3807.756 | <0.001 | 168 | 0.958 | 0.948 | 0.086 (0.084 - 0.089) |
| Multiple group ICM-CFA with 4 factors: Scalar invariance | 3868.201 | <0.001 | 190 | 0.958 | 0.953 | 0.082 (0.079 - 0.084) |
| Age | | | | | | |
| ICM-CFA with 4 factors: 18-30 | 596.666 | <0.001 | 84 | 0.934 | 0.917 | 0.105 (0.097 - 0.113) |
| ICM-CFA with 4 factors: 31-50 | 1301.346 | <0.001 | 84 | 0.949 | 0.936 | 0.096 (0.092 - 0.101) |
| ICM-CFA with 4 factors: 51-65 | 1244.419 | <0.001 | 84 | 0.963 | 0.954 | 0.085 (0.081 - 0.089) |
| ICM-CFA with 4 factors: 66-79 | 907.702 | <0.001 | 84 | 0.962 | 0.953 | 0.081 (0.076 - 0.086) |
| ICM-CFA with 4 factors: 80+ | 326.004 | <0.001 | 84 | 0.924 | 0.905 | 0.105 (0.093 - 0.117) |
| Multiple group ICM-CFA with 4 factors: Configural invariance | 4376.136 | <0.001 | 420 | 0.955 | 0.944 | 0.090 (0.088 - 0.093) |
| Multiple group ICM-CFA with 4 factors: Scalar invariance | 4796.028 | <0.001 | 508 | 0.951 | 0.950 | 0.085 (0.083 - 0.088) |
|  | ɣ² | p | df | CFI | TLI | RMSEA (90% CI) |
| **Respondents: Professionals** | | | | | | |
| Type of respondents | | | | | | |
| ICM-CFA with 4 factors: Middle management (Staff members and supervisors) | 554.788 | <0.001 | 84 | 0.940 | 0.926 | 0.079 (0.073 - 0.086) |
| ICM-CFA with 4 factors: Physicians / Dentists ( | 544.594 | <0.001 | 84 | 0.944 | 0.931 | 0.079 (0.073 - 0.085) |
| ICM-CFA with 4 factors: Nurses / Midwives /  Nursing assistants | 1815.336 | <0.001 | 84 | 0.948 | 0.935 | 0.081 (0.078 - 0.085) |
| ICM-CFA with 4 factors: Other professionals with direct patient contact | 1188.801 | <0.001 | 84 | 0.936 | 0.920 | 0.092 (0.088 - 0.097) |
| ICM-CFA with 4 factors: Supporting professionals without direct patient contact | 665.688 | <0.001 | 84 | 0.957 | 0.947 | 0.082 (0.076 - 0.088) |
| ICM-CFA with 4 factors: Management and executives | 183.163 | <0.001 | 84 | 0.910 | 0.888 | 0.092 (0.073 - 0.110) |
| Multiple group ICM-CFA with 4 factors: Configural invariance | 4952.371 | <0.001 | 504 | 0.946 | 0.932 | 0.083 (0.081 - 0.086) |
| Multiple group ICM-CFA with 4 factors: Scalar invariance | 5899.051 | <0.001 | 614 | 0.935 | 0.934 | 0.082 (0.080 - 0.084) |
| Gender | | | | | | |
| ICM-CFA with 4 factors: Female | 3427.769 | <0.001 | 84 | 0.946 | 0.933 | 0.083 (0.081 - 0.086) |
| ICM-CFA with 4 factors: Male | 869.511 | <0.001 | 84 | 0.958 | 0.948 | 0.072 (0.068 - 0.076) |
| Multiple group ICM-CFA with 4 factors: Configural invariance | 4297.280 | <0.001 | 168 | 0.949 | 0.936 | 0.081 (0.079 - 0.083) |
| Multiple group ICM-CFA with 4 factors: Scalar invariance | 4388.301 | <0.001 | 190 | 0.948 | 0.943 | 0.077 (0.075 - 0.079) |
| Age | | | | | | |
| ICM-CFA with 4 factors: 18-30 | 883.282 | <0.001 | 84 | 0.939 | 0.923 | 0.082 (0.077 - 0.087) |
| ICM-CFA with 4 factors: 31-50 | 2314.210 | <0.001 | 84 | 0.943 | 0.929 | 0.085 (0.082 - 0.088) |
| ICM-CFA with 4 factors: 51-65 | 1232.295 | <0.001 | 84 | 0.961 | 0.951 | 0.075 (0.071 - 0.079) |
| Multiple group ICM-CFA with 4 factors: Configural invariance | 4429.786 | <0.001 | 252 | 0.949 | 0.936 | 0.081 ( 0.079 - 0.083) |
| Multiple group ICM-CFA with 4 factors: Scalar invariance | 4689.450 | <0.001 | 296 | 0.946 | 0.943 | 0.077 (0.075 - 0.079) |

Notes: CFI = comparative fit index, ICM-CFA = independent cluster model confirmatory factor analysis; RMSEA = root mean square error of approximation; TLI = Tucker-Lewis index

**Supplementary Table 2** Goodness-of-fit indices associated with factor analyses for part 2 ‘Healthcare quality for professionals’

|  | ɣ² | p | df | CFI | TLI | RMSEA (90% CI) |
| --- | --- | --- | --- | --- | --- | --- |
| **Respondents: Patients and kin** | | | | | | |
| Type of respondents | | | | | | |
| ICM-CFA with 4 factors: Patients | 3067.178 | <0.001 | 84 | 0.968 | 0.960 | 0.087 (0.085 - 0.090) |
| ICM-CFA with 4 factors: Kin | 829.525 | <0.001 | 84 | 0.966 | 0.958 | 0.087 (0.082 - 0.093) |
| Multiple group ICM-CFA with 4 factors: Configural invariance | 3896.703 | <0.001 | 168 | 0.967 | 0.959 | 0.087 (0.085 - 0.090) |
| Multiple group ICM-CFA with 4 factors: Scalar invariance | 3946.495 | <0.001 | 190 | 0.967 | 0.964 | 0.082 (0.080 - 0.085) |
| Gender | | | | | | |
| ICM-CFA with 4 factors: Female | 2372.106 | <0.001 | 84 | 0.965 | 0.956 | 0.091 (0.088 - 0.094) |
| ICM-CFA with 4 factors: Male | 1483.919 | <0.001 | 84 | 0.972 | 0.965 | 0.082 (0.078 - 0.086) |
| Multiple group ICM-CFA with 4 factors: Configural invariance | 3856.025 | <0.001 | 168 | 0.968 | 0.960 | 0.087 (0.085 - 0.089) |
| Multiple group ICM-CFA with 4 factors: Scalar invariance | 3901.119 | <0.001 | 190 | 0.968 | 0.964 | 0.082 (0.080 - 0.084) |
| Age | | | | | | |
| ICM-CFA with 4 factors: 18-30 | 536.583 | <0.001 | 84 | 0.952 | 0.940 | 0.099 (0.091 - 0.107) |
| ICM-CFA with 4 factors: 31-50 | 1415.310 | <0.001 | 84 | 0.957 | 0.946 | 0.101 (0.096 - 0.105) |
| ICM-CFA with 4 factors: 51-65 | 1343.880 | <0.001 | 84 | 0.967 | 0.959 | 0.088 (0.084 - 0.093) |
| ICM-CFA with 4 factors: 66-79 | 1003.045 | <0.001 | 84 | 0.971 | 0.963 | 0.085 (0.081 - 0.090) |
| ICM-CFA with 4 factors: 80+ | 367.212 | <0.001 | 84 | 0.943 | 0.929 | 0.114 (0.102 - 0.126) |
| Multiple group ICM-CFA with 4 factors: Configural invariance | 4666.029 | <0.001 | 420 | 0.963 | 0.954 | 0.093 (0.091 - 0.096) |
| Multiple group ICM-CFA with 4 factors: Scalar invariance | 4932.223 | <0.001 | 508 | 0.962 | 0.960 | 0.087 (0.084 - 0.089) |
|  | ɣ² | p | df | CFI | TLI | RMSEA (90% CI) |
| **Respondents: Professionals** | | | | | | |
| Type of respondents | | | | | | |
| ICM-CFA with 4 factors: Middle management (Staff members and supervisors) | 566.354 | <0.001 | 84 | 0.950 | 0.937 | 0.080 (0.074 - 0.086) |
| ICM-CFA with 4 factors: Physicians / Dentists | 666.959 | <0.001 | 84 | 0.944 | 0.930 | 0.089 (0.083 - 0.095) |
| ICM-CFA with 4 factors: Nurses / Midwives /  Nursing assistants | 2759.553 | <0.001 | 84 | 0.936 | 0.920 | 0.101 (0.098 - 0.104) |
| ICM-CFA with 4 factors: Other professionals with direct patient contact | 1202.165 | <0.001 | 84 | 0.943 | 0.928 | 0.093 (0.088 – 0.098) |
| ICM-CFA with 4 factors: Supporting professionals without direct patient contact | 816.174 | <0.001 | 84 | 0.946 | 0.932 | 0.092 (0.086 – 0.098) |
| ICM-CFA with 4 factors: Management and executives | 177.876 | <0.001 | 84 | 0.908 | 0.885 | 0.089 (0.071 - 0.107) |
| Multiple group ICM-CFA with 4 factors: Configural invariance | 6189.082 | <0.001 | 504 | 0.941 | 0.926 | 0.094 (0.092 – 0.096) |
| Multiple group ICM-CFA with 4 factors: Scalar invariance | 7367.407 | <0.001 | 614 | 0.929 | 0.928 | 0.093 (0.091 – 0.095) |
| Gender | | | | | | |
| ICM-CFA with 4 factors: Female | 4328.971 | <0.001 | 84 | 0.943 | 0.929 | 0.094 (0.092 - 0.096) |
| ICM-CFA with 4 factors: Male | 1153.371 | <0.001 | 84 | 0.953 | 0.941 | 0.084 (0.080 - 0.088) |
| Multiple group ICM-CFA with 4 factors: Configural invariance | 5482.343 | <0.001 | 168 | 0.945 | 0.931 | 0.092 (0.090 – 0.094) |
| Multiple group ICM-CFA with 4 factors: Scalar invariance | 5672.258 | <0.001 | 190 | 0.943 | 0.937 | 0.088 (0.086 – 0.090) |
| Age | | | | | | |
| ICM-CFA with 4 factors: 18-30 | 1101.248 | <0.001 | 84 | 0.940 | 0.925 | 0.092 (0.087 - 0.097) |
| ICM-CFA with 4 factors: 31-50 | 2962.542 | <0.001 | 84 | 0.940 | 0.925 | .096 (.093 - .099) |
| ICM-CFA with 4 factors: 51-65 | 1699.954 | <0.001 | 84 | 0.951 | 0.938 | 0.089 (0.085 - 0.093) |
| Multiple group ICM-CFA with 4 factors: Configural invariance | 5763.745 | <0.001 | 252 | 0.944 | 0.929 | 0.093 (0.091 – 0.095) |
| Multiple group ICM-CFA with 4 factors: Scalar invariance | 6022.453 | <0.001 | 296 | 0.941 | 0.938 | 0.088 (0.086 – 0.090) |

Notes: CFI = comparative fit index, ICM-CFA = independent cluster model confirmatory factor analysis; RMSEA = root mean square error of approximation; TLI = Tucker-Lewis index.
